# Factors affecting mechanical ventilation in icu elderly patients

**DOI:** 10.1186/2197-425X-3-S1-A660

**Published:** 2015-10-01

**Authors:** E Pappa, H Pavlou, M Eforakopoulou

**Affiliations:** KAT General Hospital, ICU, Athens, Greece

## Introduction

In recent years the rate of elderly patients being hospitalized in ICU and requiring respiratory support has increased. The rapid weaning from mechanical ventilation is often difficult in these patients.

## Objectives

The aim of this study is to search for the factors that influence the duration of the elderly patients' mechanical ventilation.

## Methods

We studied retrospectively 74 patients >65 years old, who have been hospitalized in ICU and underwent mechanical ventilation. The patients, depending on the duration of the mechanical respiratory support (MRS), have been divided into two groups. Group A: MRS < 10 days and Group B: MRS >10 days. We have recorded the age, the gender, the cause of admission in ICU, the comorbidities (Charlson comorbidity index), the duration of hospitalization, the complications and the outcome. For the statistics analysis we have used t- student and chi-square tests.

## Results

We studied 43 men and 31 women with average age 79 ± 6.4 years old. The cause of admission in ICU has been trauma (26%), surgical reasons (18%) and pathological reasons (57%) and it has not been related to the length of mechanical ventilation. The increased comorbidity (Charlson comorbidity index) and the ICU complications (pneumonia, sepsis-MODS) have significantly increased the duration of mechanical ventilation (p < 0.01). The length of hospital stay and mortality were considerably greater among patients in Group B (p = 0.003 and p = 0.0006 respectively).

## Conclusions

The comorbidities and the complications during ICU hospitalization increase the duration of mechanical respiratory support in elderly patients. The length of hospital stay and the mortality are increased in these patients.
